# Book Notices

**Published:** 1869-03

**Authors:** 


### Book Notices.

Practical Observations on the Etiology, Pathology, Diagnosis, and Treatment of Anal Fissure. By WILLIAM BODENHAMER, A.M., M.D., Professor of the Diseases, Injuries, and Malformations of the Rectum, Anus, and Genito-Urinary Organs. Illustrated by numerous Cases and Drawings. New York: William Wood & Co., 61 Walker Street. 1868. 199 pages. Price, $2.25.

---

Essentials of the Principles and Pactice of Medicine: A Hand-Book for Students and Practitioners. By HENRY HARTSHORNE, M.D., Professor of Hygiene in the University of Pennsylvania, etc., etc., etc. Second Edition, revised and improved. Philadelphia: Henry C. Lea. 1869.

This is a small-sized octavo, of 425 pages, containing an admirably-condensed summary of the principles and practice of medicine. We have always doubted the real utility of such works. That they are a great convenience to students and practitioners of doubtful industry, there can be no doubt; and, as there are many such in the profession, there is an active demand for such works.

We do not hesitate to say that this work of Dr. Hartshorne is the very best of its kind.

---

We have also received from the well-known publishing house of Henry C. Lea, of Philadelphia, through the hands of Cobb, Pritchard & Co., of this city, the following work:—

Clinical Lectures on Diseases of the Urinary Organs: Delivered at the University College Hospital. By Sir HENRY THOMPSON, Surgeon Extraordinary to H. M. the King of the Belgians; Professor of Clinical Surgery; and Surgeon to the University College Hospital. With Illustrations. Philadelphia: Henry C. Lea. 1869. 204 pages.

The volume contains twelve Lectures on the following topics:—First, Introductory Diagnosis; second and third, Stricture of the Urethra; fourth, Hypertrophy of the Prostate, and its Consequences; fifth, Retention of Urine; sixth, Extravasation of Urine and Urinary Fistulæ; seventh, Stone in the Bladder; eighth, Lithotrity; ninth, Lithotomy; tenth, Cystitis and Prostatitis; eleventh, Diseases of the Bladder, Paralysis, Atony, Juvenile Incontinence, Tumors; twelfth, Hæmaturia, and Renal Calculus.

---

A Conspectus of the Medical Sciences: Containing Manuels of Anatomy, Physiology, Chemistry, Materia Medica, Practice of Medicine, Surgery, and Obstetrics, for the Use of Students. By HENRY HARTSHORNE, A.M., M.D., Professor of Hygiene in the University of Pennsylvania, Auxiliary Faculty of Medicine, etc., etc., etc. With 310 Illustrations. Philadelphia: Henry C. Lea. 1869.

In this work Dr. Hartshorne has endeavored to do for all the ordinary branches of medical science what he had accomplished for practical medicine alone, in the work just noticed. In this conspectus we have a carefully condensed summary of all the branches named above, in one volume of 1002 pages. Both works are for sale by Cobb, Pritchard & Co., 81 Lake St.

---

A Hand-Book on Uterine Therapeutics, and of Diseases of Women. By EDWARD JOHN TILT, M.D., Member of the Royal College of Physicians; Consulting Physician to the Farringden General Dispensary; Fellow of the Royal Medical Chirurgical Society; etc., etc., etc. Second American Edition; thoroughly Revised and Amended. D. Appleton & Co., 90, 92, and 94 Grand Street, New York. 1869.

This is a full sized octavo volume of 345 pages, published in good style, except the type are too small for pleasent reading. The merits of the author are well known; and this edition of his work ought to be in the hands of every practitioner; for the special purpose of counteracting the modern tendency to reduce uterine therapeutics to the knife, the caustic, and mechanical appliances. We commend it to our readers as a book worth buying. For sale by S. C. Griggs & Co., Chicago.

